# Supplementary material for: Multi-molecular hyperspectral PRM-SRS microscopy
Source: Nat Commun. 2024 Feb 21;15:1599. doi: 10.1038/s41467-024-45576-6 (PMC10881988; doi:10.1038/s41467-024-45576-6)
Supplement: Supplementary file 1 — Supplementary Information [file 41467_2024_45576_MOESM1_ESM.pdf]

Supplementary Information

Multi-molecular hyperspectral PRM-SRS microscopy

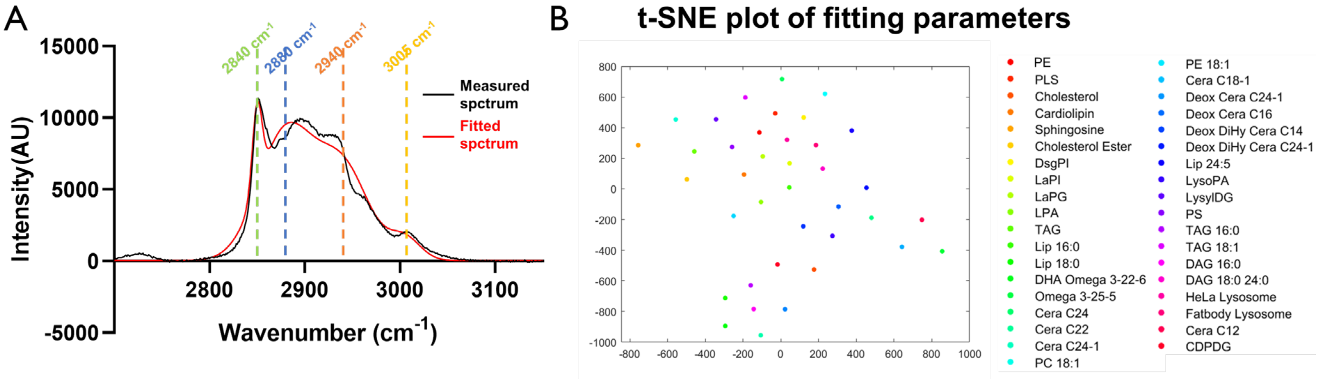

Supplementary Figure 1: CH stretching region spectrum and t-SNE plot of their fitting parameters. A) Spontaneous Raman spectrum of PE lipid. With four Gaussian peaks centered on four different wavenumbers, spectral shape was fitted. The fitting parameters, sigma and amplitude of each Gaussian peak were summarized in supplementary Table 2. **B)** To show the capability of lipid subtype separation based on CH stretching region spectra, t-SNE plot of 38 lipid subtypes is presented. The plot shows enough distances between each lipid subtype. Source data are provided as a Source Data file.

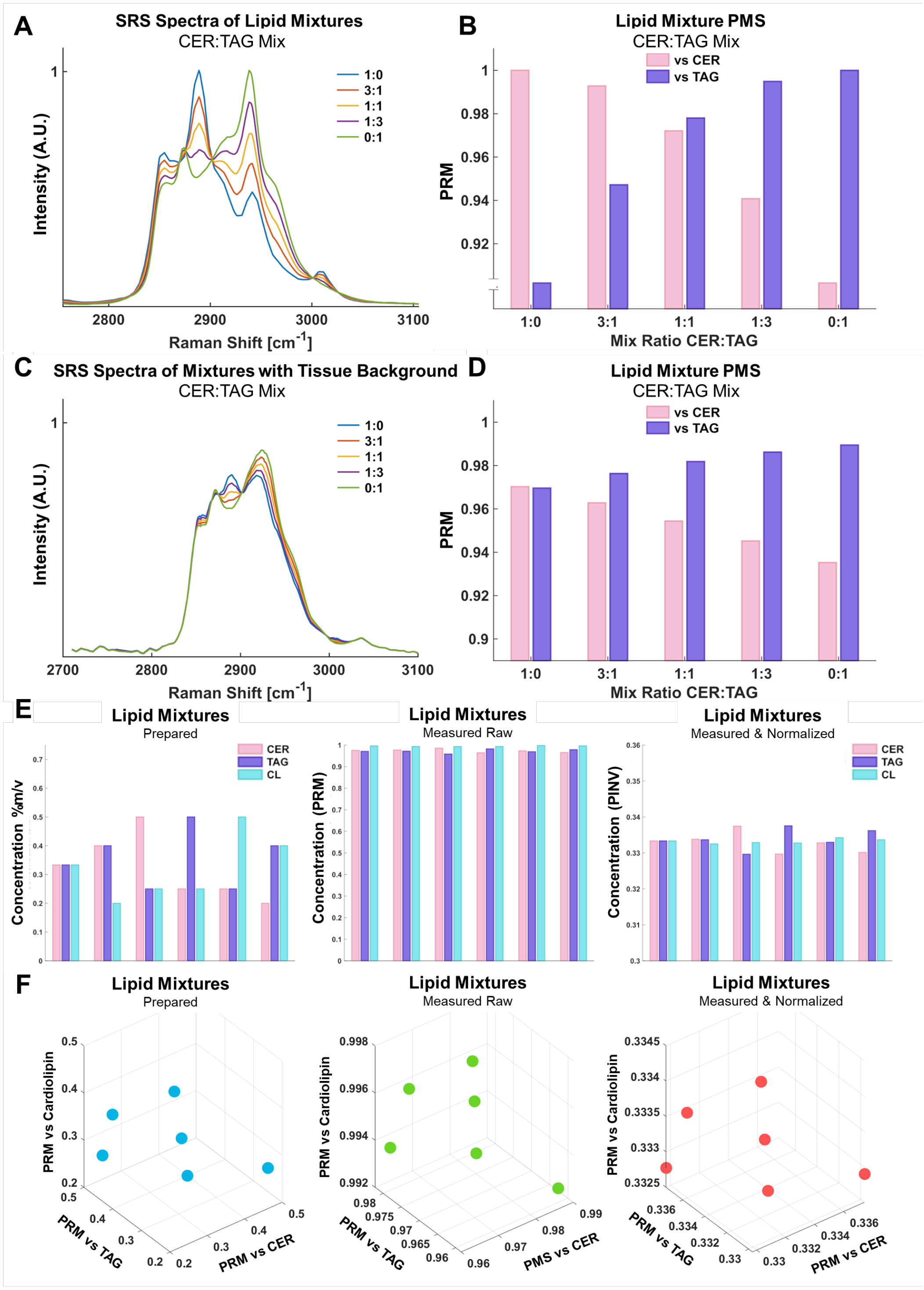

Supplementary Figure 2: Relative concentrations and similarity scores in samples containing mixtures of different lipid subtypes. A-B) Spontaneous Raman spectra and corresponding similarity scores of mixtures of ceramide and TAG (C2-C10) (Sigma 17810-1AMP-S) at the indicated ratios. **C-D)** The experiment repeated with the addition of equal volume of methanol-washed tissue lysates, showing a deterioration of similarity scores but a preservation of trend. **E)** Experiments with three-lipid mixes (CER, TAG and cardiolipin) at various ratios were also conducted. Raw similarity scores (middle panel) and relative similarity scores normalized to the first group with equal ratios (right panel) are shown. **F)** Plotting

both raw and normalized (relative) similarity scores using each component as an axis, show similar patterns to the actual concentrations. All spectra were acquired on the same instrument (HORIBA XploRa plus). The highest similarity scores were achieved with a spectral shift of 0. Source data are provided as a Source Data file.

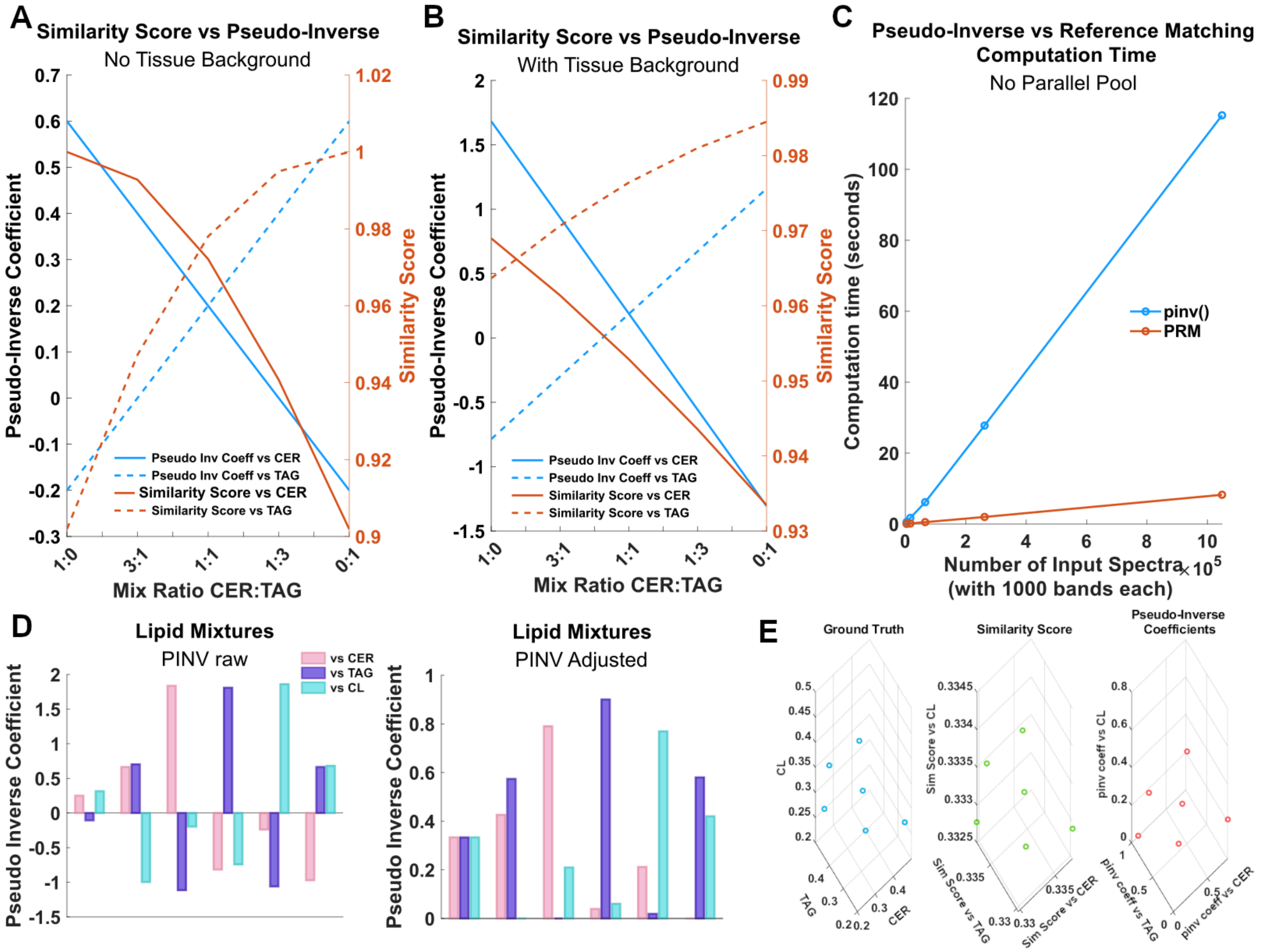

Supplementary Figure 3: Comparison of PRM and pseudo-inverse matrix multiplication. (A) The data shown in Supplementary Fig. 1 A-B were analyzed using pseudo-inverse (PINV) matrix multiplication and the resulting coefficients were plotted in blue. The similarity scores obtain by PRM were in orange. Pseudo-Inverse matrix coefficients have a linear relationship with relative ratios of the components but have negative coefficients and a theoretically unbounded range. **(B)** The data shown in Supplementary Fig. 1C-D were analyzed using PINV and the resulting coefficients were plotted (blue) against those using PRM (orange). PINV shows a better linear relationship than PRM similarity scores but retains the negative and unbounded range. **(C)** Comparison of computation time for PRM and PINV with single processing unit and no spectral shifts shows that PINV takes much longer time to compute compared with PRM. PINV is also a one-liner that is difficult to split to parallel processing units, and does not allow for the input matrix to be split into more manageable sizes because the rank sums would not be equal. **(D)** The data shown in Supplementary Fig 1E-F were re-analyzed using PINV, and the linear relationship is also observed, but additional rescaling steps removing negative values were required to obtain concentration-corelated results. **(E)** The three-lipid mixtures from (D) are plotted in 3D, showing that both PRM and PINV can obtain results similar to the ground truth. Source data are provided as a Source Data file.

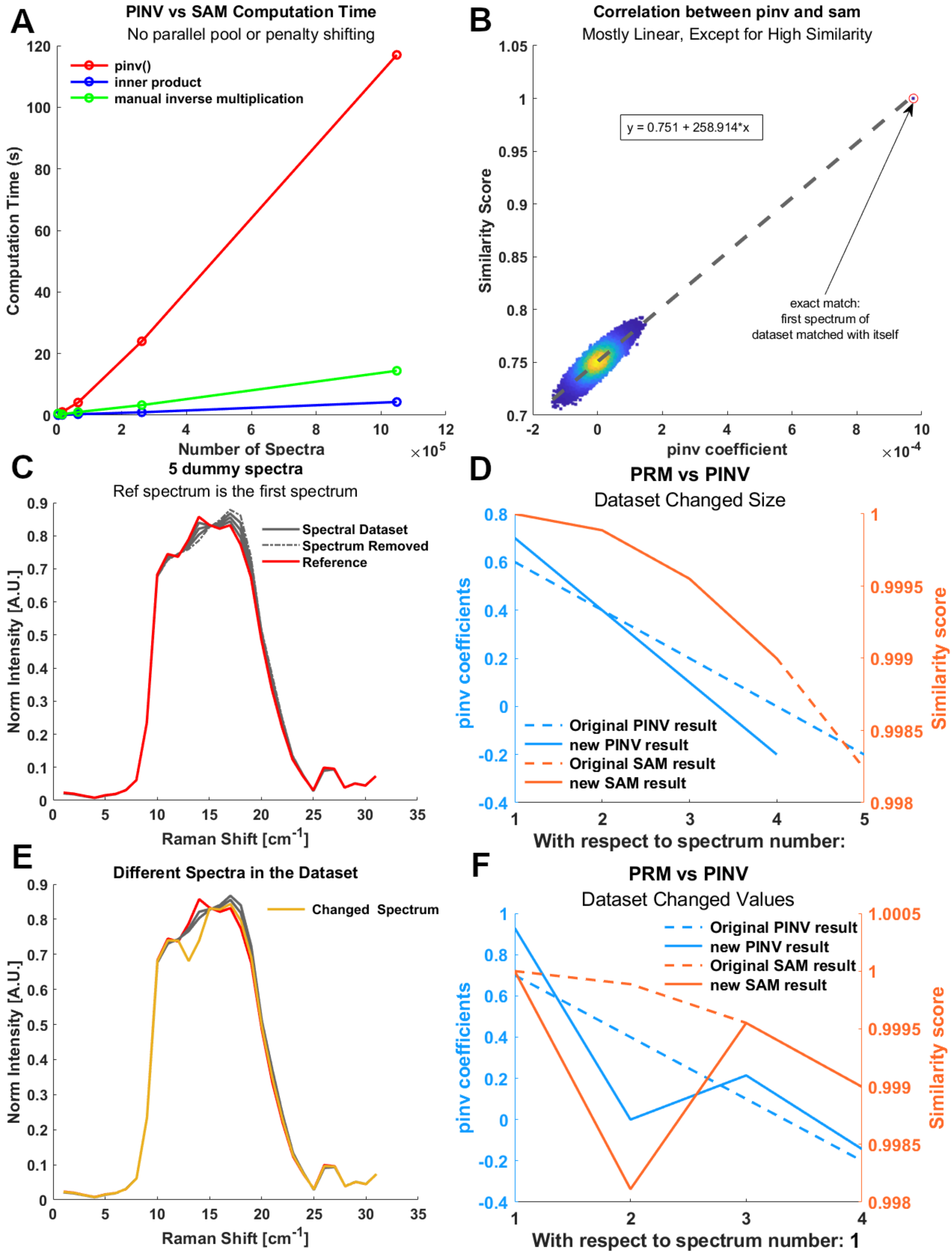
A
PINV vs SAM Computation Time
No parallel pool or penalty shifting
pinv()
inner product
manual inverse multiplication
Computation Time (s)
Number of Spectra
$\times 10^5$
B
Correlation between pinv and sam
Mostly Linear, Except for High Similarity
y = 0.751 + 258.914*x
exact match:
first spectrum of
dataset matched with itself
Similarity Score
pinv coefficient
$\times 10^{-4}$
C
5 dummy spectra
Ref spectrum is the first spectrum
Spectral Dataset
Spectrum Removed
Reference
Norm Intensity [A.U.]
Raman Shift [cm^{-1}]
D
PRM vs PINV
Dataset Changed Size
Original PINV result
new PINV result
Original SAM result
new SAM result
pinv coefficients
Similarity score
With respect to spectrum number:
E
Different Spectra in the Dataset
Changed Spectrum
Norm Intensity [A.U.]
Raman Shift [cm^{-1}]
F
PRM vs PINV
Dataset Changed Values
Original PINV result
new PINV result
Original SAM result
new SAM result
pinv coefficients
Similarity score
With respect to spectrum number: 1

Supplementary Figure 4: PRM is more robust than PINV across spectral datasets. (A) Computation time is recorded for SAM via inner product, PINV using the stock MATLAB function as of R2022b, and PINV via manual inverse matrix multiplication using the same spectral dataset for each method. The dataset consists of random integer spectra with 1000 wavenumbers each. The number of spectra computed (x-axis) in the dataset are typical of HSI sizes such as 512x512 pixel images. **(B)** The resulting similarity scores from SAM and coefficients from PINV are plotted against each other for the random dataset. The results for PINV whether via the stock MATLAB function or via manual inverse matrix multiplication are identical, so only one set is plotted. The outlying point is the result of the first spectrum in the dataset being compared to itself. Notice how the SAM result of this exact match is 1, which is intuitive, while the majority of computation results are centered at 0 for the PINV, which is also intuitive. Therefore there is a tradeoff between whether the perfect match or random match should be intuitive. Since the Raman spectra of biological samples are not entirely random, but share general similarities, SAM scores are preferred due to their intuitive range. **(C-D)** 5 dummy spectra are created and both similarity scores and PINV scores are plotted. Then one of the 5 dummy spectra is removed, and both sets of scores are re-plotted. The similarity scores via SAM of all the other spectra are unchanged, which should be expected. However, the PINV coefficients of the other spectra did change. This is critical because HSI of various sizes and pixel densities may yield different PINV coefficients even though the spectra of the analyte is exactly the same. **(E)** Adjustment one wavenumber of a spectrum only affects the similarity score of that spectrum, which should be expected. However, adjusting just one wavenumber of one spectrum in the dataset affects all other PINV coefficients in that dataset, plotted in **(F)**. Source data are provided as a Source Data file.

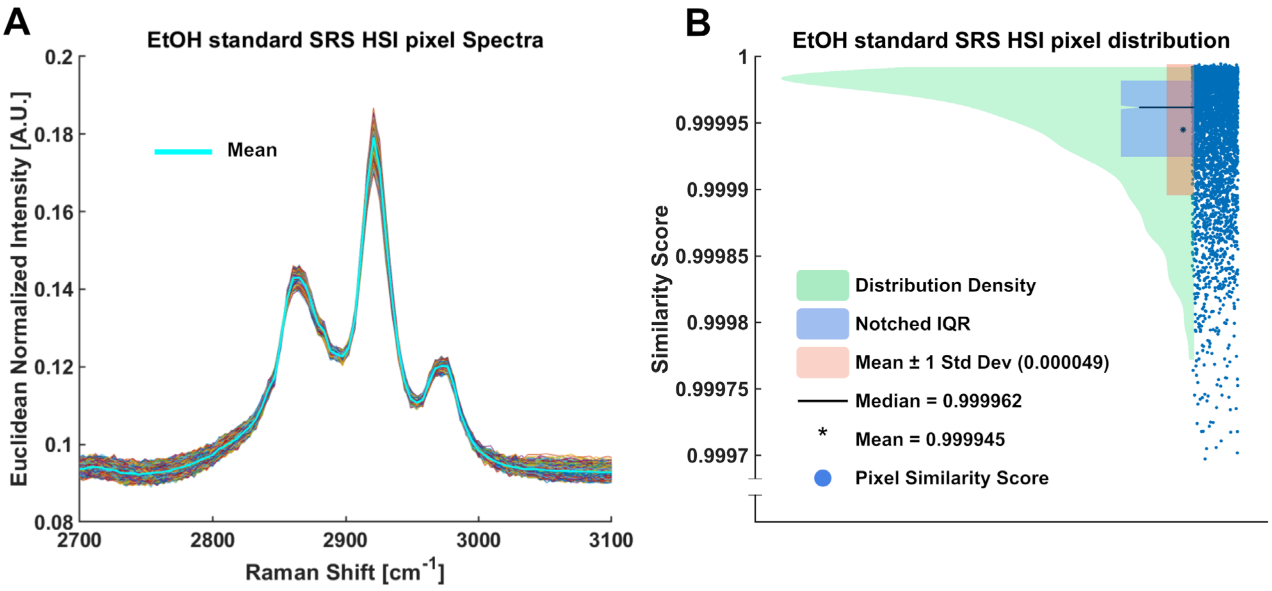

Supplementary Figure 5: 200 proof Ethanol SRS HSI spectra. (A) All pixel spectra in a 64x64 (total 4096 spectra) test SRS HSI of pure ethanol shows very little pixel-wise "noise". All pixels in the image have a relatively consistent spectral profile, as expected. **(B)** This homogeneity is translated into similarity scores, and confirms that the image quality of the SRS HSI is sufficient, considering the vast majority of pixels all have nearly perfect similarity scores with respect to a spontaneous Raman spectrum of the ethanol standard. Source data are provided as a Source Data file.

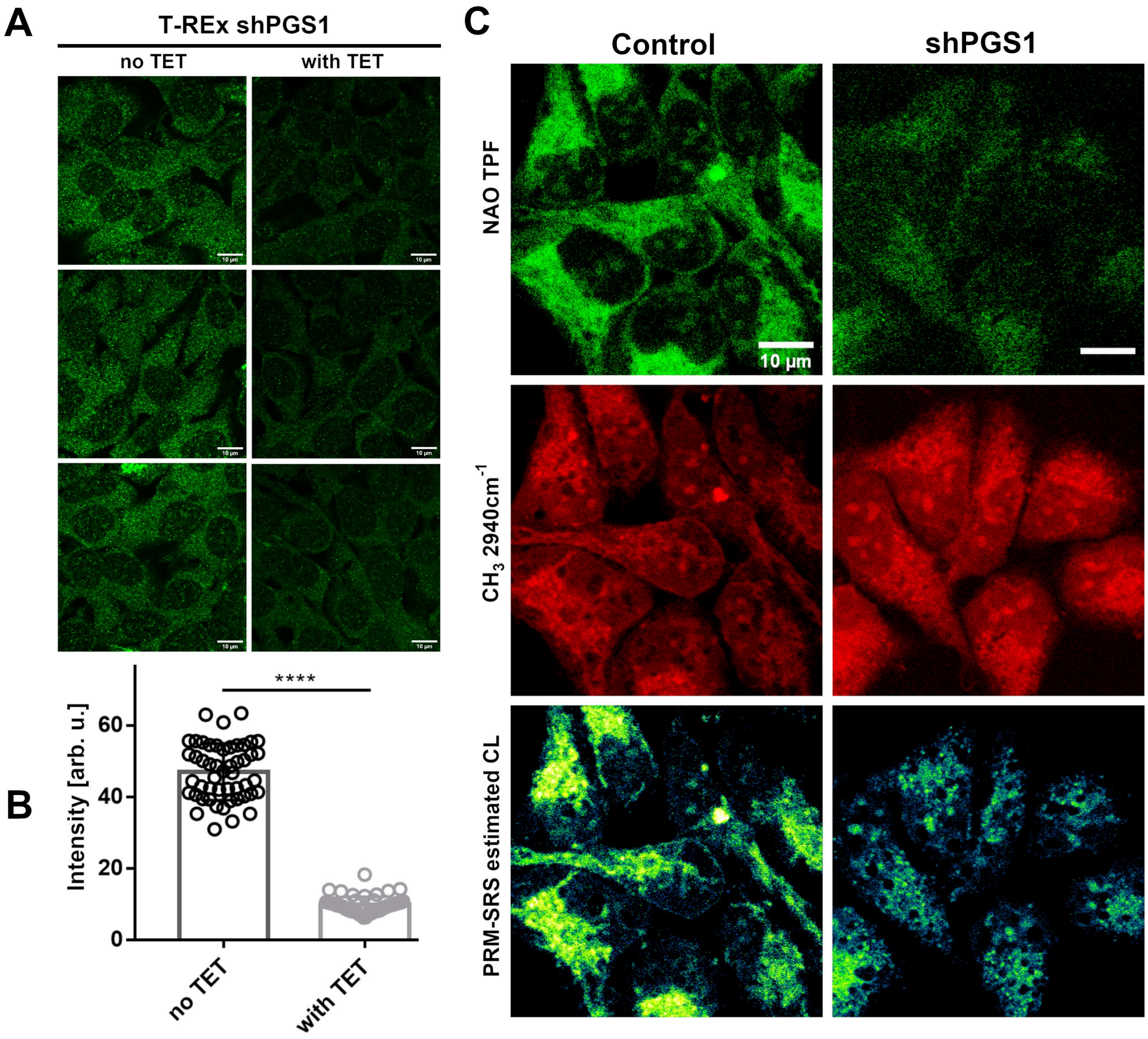

Supplementary Figure 6: Immunofluorescence microscopy confirmed reduced PGS1 protein expression in shPGS1 cells. **(A)** Immunofluorescence staining of stable shPGS1 cells using the specific PGS1 antibody. (n=50 cells in each group) **(B)** Quantification of immunofluorescence signal intensity in images shown in panel A. Compared to the control group, the PGS1 protein level was significantly reduced by shPGS1 following induction with tetracycline (Tet). **(C)** TPF (following NAO staining), SRS, and PRM-SRS images from Fig. 4 demonstrate that the similarity score-based PRM-SRS image for CL distribution is similar to that shown by NAO staining (TPF) but distinct from single Raman shift SRS images. Scale bar, 10 μm. Source data are provided as a Source Data file.

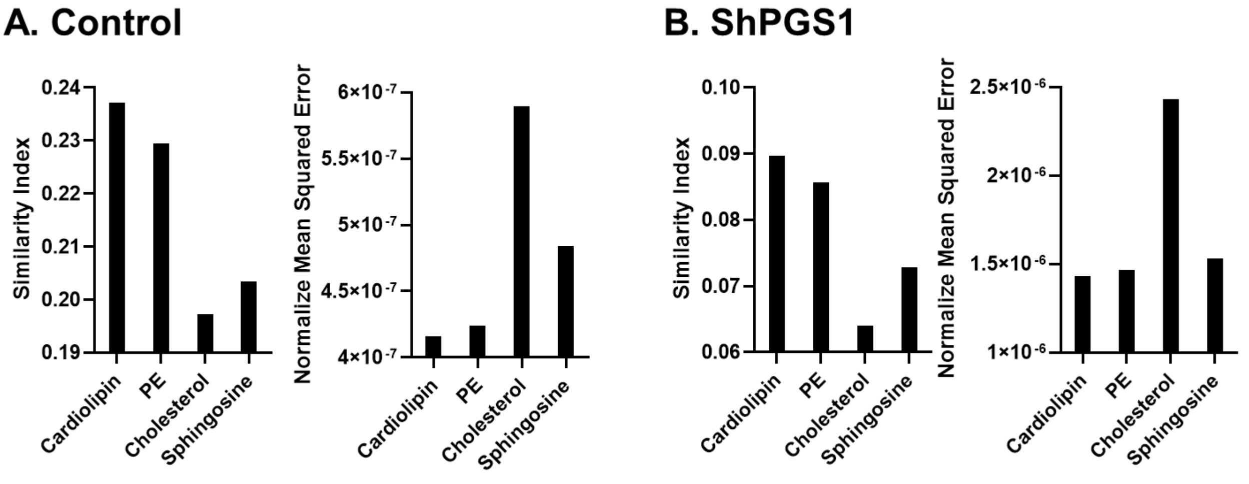

Supplementary Figure 7: Similarity indices and normalized mean squared errors between NAO stained cardiolipin image and PRM-SRS detected lipid subtype images (cardiolipin, PE, cholesterol, and sphingosine). In the two samples (**(A)**: Control, **(B)**: ShPGS1), the similarity index of cardiolipin was higher than the indices of other lipid subtypes. Normalized mean squared error of cardiolipin image was lower than other lipid subtypes. Due to the low intensity in ShPGS1 sample, similarity index was lower than control, and normalized mean squared error was higher than control. Source data are provided as a Source Data file.

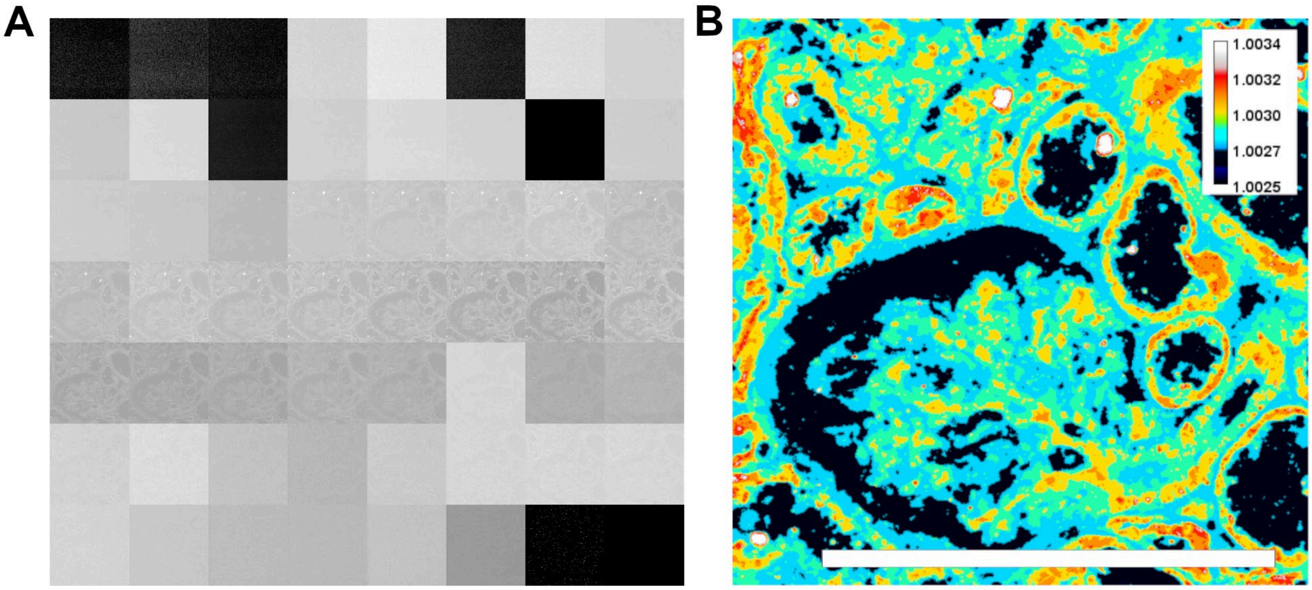

Supplementary Figure 8: HSI and Ratiometric image of Free cholesterol to Esterified Cholesterol PRM-SRS images from Figure 5. (A) Hyperspectral image frames of the human kidney tissue section from 2750 cm^{-1} to 3050 cm^{-1}. **(B) Cholesterol:cholesterol ester ratiometric image.** Ratio values are based on the similarity scores from the PRM-SRS images. Higher similarity scores for free cholesterol occur intracellularly in tubules and glomerular epithelial cells that line the arteriole and capillaries of the mesangium, as well as the larger deposits indicated by solid arrows in Figure 5B. Scale bar, 200 μm.

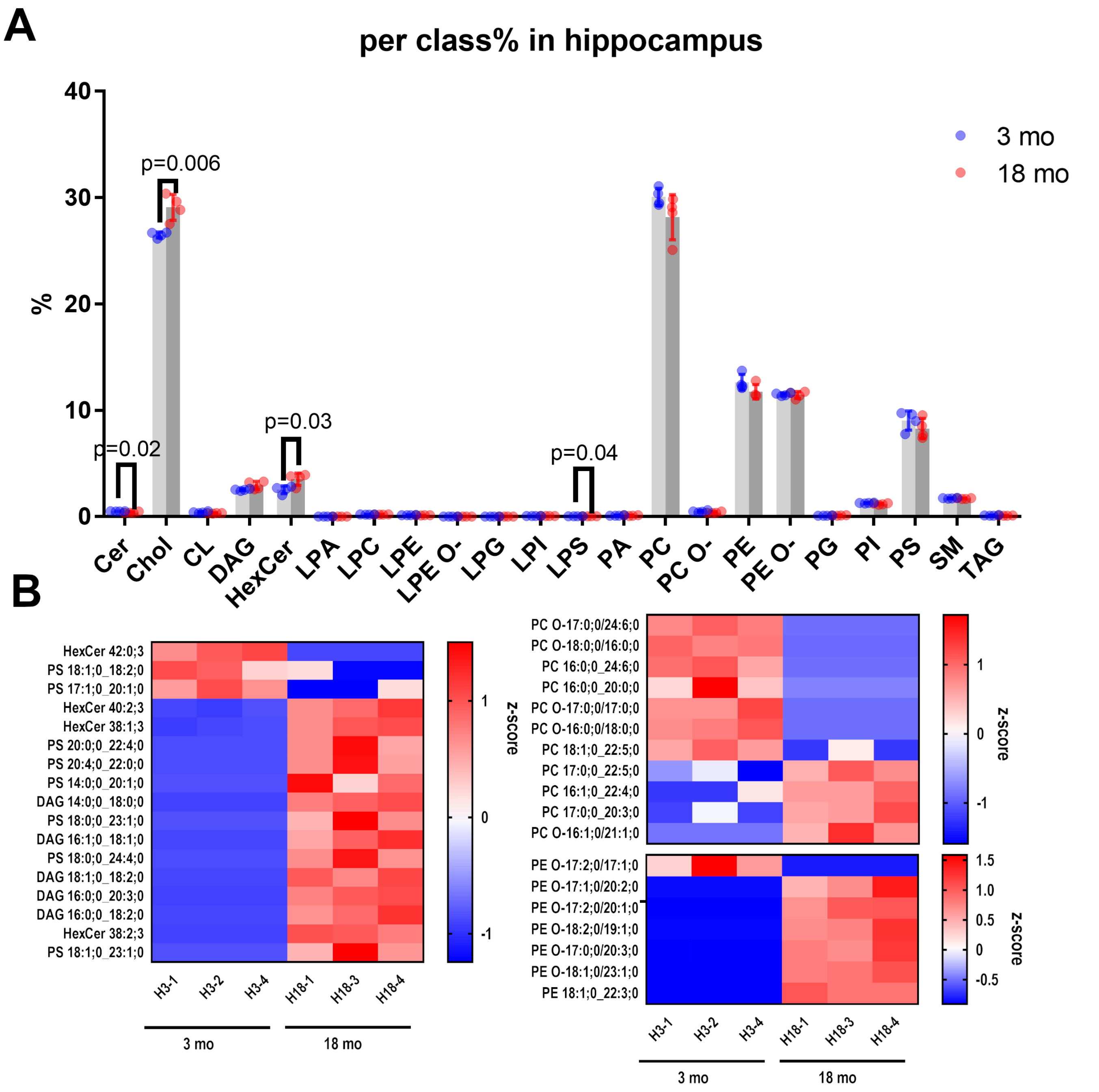

Supplementary Figure 9: Supplemental mass spectrometry results for various lipid subtypes. (n=3 in each group) **(A)** Simplex-normalized lipid concentrations. **(B)** Heatmaps generated by running against internal lipid standards show significantly changed lipids. One sample from each group is removed due to outlying data in several lipid subtypes.

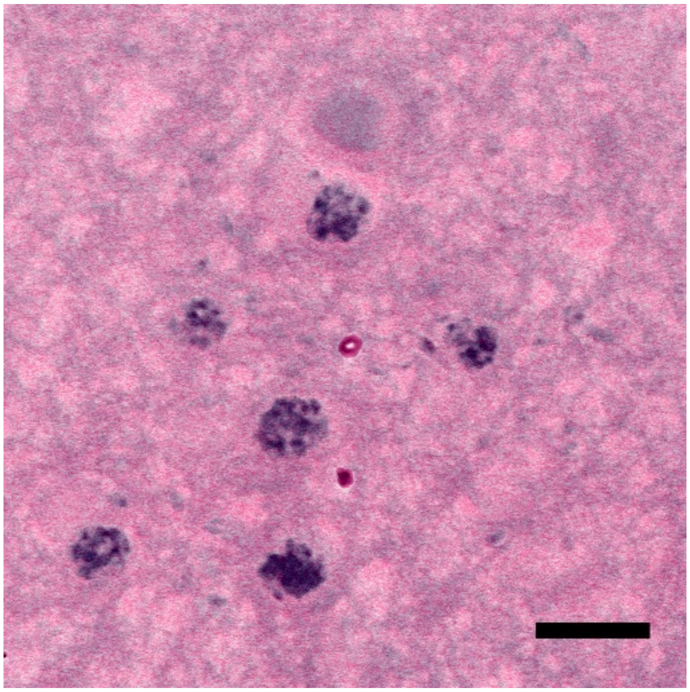

Supplementary Figure 10: SRH (virtual H&E) image of the human brain temporal cortex sample. Scale bar, 10 µm.

	C12 Ceramide	PE	PC	Cholesterol	Cholesterol Ester	TAG	Cardiolipin	Sphingosine
**C12 Ceramide**	**1**	0.9396	0.9504	0.9324	0.9415	0.8536	0.9229	**0.9927**
**PE**	0.9396	**1**	**0.9953**	0.973	0.9854	0.9497	**0.9932**	0.9155
**PC**	0.9504	**0.9953**	**1**	0.9728	0.9851	0.9437	**0.9904**	0.9277
**Cholesterol**	0.9324	0.973	0.9728	**1**	**0.9941**	0.9658	0.9725	0.8978
**Cholesterol Ester**	0.9415	0.9854	0.9851	**0.9941**	**1**	0.9604	0.9838	0.9112
**TAG**	0.8536	0.9497	0.9437	0.9658	0.9604	**1**	0.9638	0.8083
**Cardiolipin**	0.9229	**0.9932**	**0.9904**	0.9725	0.9838	0.9638	**1**	0.8903
**Sphingosine**	**0.9927**	0.9155	0.9277	0.8978	0.9112	0.8083	0.8903	**1**

Supplementary Table 1: Inner product values of 8 different lipid subtypes. The inner products of 8 different lipid subtypes show the capability of quantitative analysis. The lipid subtype pairs having high structural similarities, for example, PC and PE show very high inner product value. On the contrary, the lipid subtype pairs having low structural similarities, such as, Cholesterol and Sphingosine, have lower inner product values than other lipid subtype pairs.

	PE	PLS	Cholesterol	Cardiolipin	Sphingosine	Cholesterol Ester	DsgPI	LaPI	LaPG	LPA
2840_Amp	0.646422	0.210139	0.093545	0.385544	0.407072	0.534452	0.637567	0.518231	0.484989	0.478832
2840_Sig	3.571	2.698	11.88	3.622	5.623	8.614	3.517	3.865	4.113	3.869
2880_Amp	1	1	1	1	1	1	1	1	1	1
2880_Sig	20.31	20.86	19.05	21	17.91	19.31	20.76	20.53	20.15	20.09
2940_Amp	0.710729	1.724821	1.344092	0.785591	0.263582	0.925578	0.643712	0.572187	0.535593	0.4749
2940_Sig	19.21	18.94	20.16	18.02	10.85	19.1	21.09	17.48	17.59	19.73
3005_Amp	0.170593	0.196365	0	0.312737	0.018962	0.055071	0.121273	0.158748	0.133565	0.198126
3005_Sig	11.62	11.96	0	10.38	30	17.55	8.622	10.89	11.78	6.676

	PC	TAG	Lip_16:0	Lip_18:0	DHA_Omega_3-22-6	Omega_3-25-5	Cera_C24	Cera_C22	Cera_C24-1	PC_18:1
2840_Amp	0.616895	0.246094	0.735662	0.753657	0.004776	0.159545	0.597738	0.819062	0.610301	0.501554
2840_Sig	3.654	3.817	5.974	5.948	30	2.91	4.924	6.785	5.097	3.964
2880_Amp	1	1	1	1	1	1	1	1	1	1
2880_Sig	21.01	17.49	6.822	7.126	23.97	22.98	17.43	7.204	18.64	22.66
2940_Amp	0.662991	1.386402	0.242621	0.250994	1.005015	0.759629	0.262598	0.237303	0.386831	0.614221
2940_Sig	19.06	16.33	21.9	22.24	14.33	14.32	10.52	20	12.38	21.64
3005_Amp	0.218146	0.145165	0	0	1.124904	0.67571	0.028397	0.00704	0.130717	0.170051
3005_Sig	17.26	14.69	0	0	17.23	15.21	9.294	3.787	16.41	12.79

	PE_18:1	Cera_C18-1	Deox_Cera_C24-1	Deox_Cera_C16	Deox_DiHy_Cera_C14	Deox_DiHy_Cera_C24-1	Lip_24:5	LysoPA	LysylDG	PS
2840_Amp	0.488277	0.287382	0.789593	0.500119	0.500119	0.494755	0.159589	0.594091	0.622972	0.582002
2840_Sig	3.79	13.12	5.972	4.078	4.078	3.989	2.909	4.935	4.994	3.534
2880_Amp	1	1	1	1	1	1	1	1	1	1
2880_Sig	22.31	18.67	8.196	16.68	16.68	16.84	22.99	15	15.58	19.61
2940_Amp	0.571619	0.408259	0.252	0.266428	0.266428	0.257381	0.759561	0.284488	0.493822	0.569123
2940_Sig	21.81	11.81	22.69	13.8	13.8	12.12	14.32	15.52	18.59	17.91
3005_Amp	0.197337	0.110807	0.038711	0.07336	0.07336	0.055314	0.675759	0.019894	0.04365	0.211444
3005_Sig	5.941	11.75	3.948	4.338	4.338	2.738	15.21	5.417	14.37	12.85

	TAG_16:0	TAG_18:1	DAG_16:0	DAG_18:0_24:0	HeLa_Lysosome	Fatbody_Lysosome	Cera_C12	CDPDG
2840_Amp	0.737984	0.586962	0.75	0.506423	0.347758	0.42752	0.287382	0.709225
2840_Sig	5.974	3.653	5.447	3.347	5	3.304	13.12	4.886
2880_Amp	1	1	1	1	1	1	1	1
2880_Sig	8.857	20.78	9.169	20.96	20.96	20.4	18.67	14.39
2940_Amp	0.277126	0.633666	0.234547	0.697421	0.868214	0.895266	0.408259	0.484391
2940_Sig	20.63	16.35	22.04	16.03	18.49	17.08	11.81	20.63
3005_Amp	0	0.16755	0	0.241491	0.177682	0.14729	0.110807	0.008947
3005_Sig	0	5.325	0	11.2	9.951	9.398	11.75	3.867

Supplementary Table 2: Fitting parameters of four Gaussian curves to describe spectral shape. Fitting parameters to define shapes of four Gaussian peaks, amplitudes and sigma values, are listed. Based on this information t-SNE plot was prepared. Source data are provided as a Source Data file.
