# Supplementary material for: Multi-molecular hyperspectral PRM-SRS microscopy
Source: Nat Commun. 2024 Feb 21;15:1599. doi: 10.1038/s41467-024-45576-6 (PMC10881988; doi:10.1038/s41467-024-45576-6)
Supplement: Supplementary file 3 — Reporting Summary [file 41467_2024_45576_MOESM3_ESM.pdf]

nature portfolio

Corresponding author(s): Lingyan Shi

Last updated by author(s): Jan 18, 2024

Reporting Summary

Nature Portfolio wishes to improve the reproducibility of the work that we publish. This form provides structure for consistency and transparency in reporting. For further information on Nature Portfolio policies, see our Editorial Policies and the Editorial Policy Checklist.

Statistics

For all statistical analyses, confirm that the following items are present in the figure legend, table legend, main text, or Methods section.

n/a	Confirmed	
☐	☒	The exact sample size (*n*) for each experimental group/condition, given as a discrete number and unit of measurement
☐	☒	A statement on whether measurements were taken from distinct samples or whether the same sample was measured repeatedly
☐	☒	The statistical test(s) used AND whether they are one- or two-sided<br>*Only common tests should be described solely by name; describe more complex techniques in the Methods section.*
☒	☐	A description of all covariates tested
☒	☐	A description of any assumptions or corrections, such as tests of normality and adjustment for multiple comparisons
☐	☒	A full description of the statistical parameters including central tendency (e.g. means) or other basic estimates (e.g. regression coefficient) AND variation (e.g. standard deviation) or associated estimates of uncertainty (e.g. confidence intervals)
☐	☒	For null hypothesis testing, the test statistic (e.g. *F*, *t*, *r*) with confidence intervals, effect sizes, degrees of freedom and *P* value noted<br>*Give P values as exact values whenever suitable.*
☒	☐	For Bayesian analysis, information on the choice of priors and Markov chain Monte Carlo settings
☒	☐	For hierarchical and complex designs, identification of the appropriate level for tests and full reporting of outcomes
☒	☐	Estimates of effect sizes (e.g. Cohen's *d*, Pearson's *r*), indicating how they were calculated

Our web collection on statistics for biologists contains articles on many of the points above.

Software and code

Policy information about availability of computer code

Data collection	Spectral data were collected using LabSpec6 v6.5.1.24, hyperspectral and multimodal images were collected using FV3000SW
Data analysis	Data were analyzed and plotted using MATLAB R2021b or later, with custom scripts for PRM-SRS, which will be made publicly available. Some images were also displayed using ImageJ and its stock LUTs and plotting plugins. We used the newly developed PRM-SRS code based on python, we published it in Github. https://github.com/lingyanshi2020/PRM-SRS

For manuscripts utilizing custom algorithms or software that are central to the research but not yet described in published literature, software must be made available to editors and reviewers. We strongly encourage code deposition in a community repository (e.g. GitHub). See the Nature Portfolio guidelines for submitting code & software for further information.

Data

Policy information about availability of data

All manuscripts must include a data availability statement. This statement should provide the following information, where applicable:

- Accession codes, unique identifiers, or web links for publicly available datasets
- A description of any restrictions on data availability
- For clinical datasets or third party data, please ensure that the statement adheres to our policy

The data that support the findings of this study are available from the corresponding author upon reasonable request.

The PRM-SRS code may be available to the public upon request to the authors. All the data supporting the findings of this study are available within the paper and its supplementary information files.

Research involving human participants, their data, or biological material

Policy information about studies with human participants or human data. See also policy information about sex, gender (identity/presentation), and sexual orientation and race, ethnicity and racism.

Reporting on sex and gender	Sex and gender are not considered in this study because of the limitation of sample size.
Reporting on race, ethnicity, or other socially relevant groupings	N/A
Population characteristics	N/A
Recruitment	N/A
Ethics oversight	N/A

Note that full information on the approval of the study protocol must also be provided in the manuscript.

Field-specific reporting

Please select the one below that is the best fit for your research. If you are not sure, read the appropriate sections before making your selection.

☒ Life sciences ☐ Behavioural & social sciences ☐ Ecological, evolutionary & environmental sciences

For a reference copy of the document with all sections, see nature.com/documents/nr-reporting-summary-flat.pdf

Life sciences study design

All studies must disclose on these points even when the disclosure is negative.

Sample size	As an exploratory study demonstrating a new algorithm, no sample size calculations were conducted a priori.<br>Cell Culture: 3 passages, 15 regions of interest each.<br>Kidney: 1 patient each (Diabetic and Control), 3 regions of interest each<br>Drosophila: 2 replicates of 5 flies in each generation, 2 regions of interest per drosophila fatbody<br>mouse brain: 3 mice in each generation, 3 regions of interest in each segment (dentate gyrus, and cortex)
Data exclusions	no data was excluded in analyses, but representative images were selected for display
Replication	For tissue samples derived from patients such as human kidney biopsies, there was no replication, but various regions of interests were taken. For mice brains, various regions in various mice brains were taken, and 3 groups of mice was used. For cell culture, and drosophila dissections, triplicate passages and generations, respectively, were analyzed.
Randomization	The covariates is not relevant to those experiments, because the read out we concern was direct visualization of spatial distribution from newly synthesized lipid. Every specimen was used. regions of interest were not randomized because visual inspection is necessary to capture a region of interest.
Blinding	Blinding was not needed to this study. The images and the algorithms cannot be biased in this study. Therefore, blinding experiments are not applicable in this case. Sample preparation such as cell culture entail no cross contamination, and therefore knowledge of the cell lines. Additionally, image and spectral acquisition and analyses are all conducted by the same group, and we knew which samples were which during analysis.

Reporting for specific materials, systems and methods

We require information from authors about some types of materials, experimental systems and methods used in many studies. Here, indicate whether each material, system or method listed is relevant to your study. If you are not sure if a list item applies to your research, read the appropriate section before selecting a response.

Materials & experimental systems

n/a	Involved in the study
☐	☒ Antibodies
☐	☒ Eukaryotic cell lines
☒	☐ Palaeontology and archaeology
☐	☒ Animals and other organisms
☒	☐ Clinical data
☒	☐ Dual use research of concern
☒	☐ Plants

Methods

n/a	Involved in the study
☒	☐ ChIP-seq
☒	☐ Flow cytometry
☒	☐ MRI-based neuroimaging

Antibodies

Antibodies used	Rabbit anti-PGS1 and secondary antibody conjugated with Alexa-488.
Validation	vendor website please check 1). Rabbit anti-PGS1(Sigma-Aldrich, Cat# AV48896) and 2).secondary antibody conjugated with Alexa-488 (Abcam, Cat# ab150081).

Eukaryotic cell lines

Policy information about cell lines and Sex and Gender in Research

Cell line source(s)	T-REx-293 Cell Line
Authentication	T-REx-293 does not authenticate the cells.
Mycoplasma contamination	Cell lines were not tested for mycoplasma contamination.
Commonly misidentified lines (See ICLAC register)	No commonly misidentified cell lines were used.

Animals and other research organisms

Policy information about studies involving animals; ARRIVE guidelines recommended for reporting animal research, and Sex and Gender in Research

Laboratory animals	Drosophila (wild type, w1118 stock #5905, 7-day old)
Wild animals	No wild animals are used in the study
Reporting on sex	We are looking at the larval stages, sex is not considered in this study
Field-collected samples	The line was brought from the stock center. No field collected samples are used in the study.
Ethics oversight	Experimental protocols are approved by the Institutional Animal Care and Use Committee (IACUC) at University of California San Diego, approved protocol number is S20123

Note that full information on the approval of the study protocol must also be provided in the manuscript.
