# Supplementary figures and images for: Multi-molecular hyperspectral PRM-SRS microscopy
Source: Nat Commun. 2024 Feb 21;15:1599. doi: 10.1038/s41467-024-45576-6 (PMC10881988; doi:10.1038/s41467-024-45576-6)

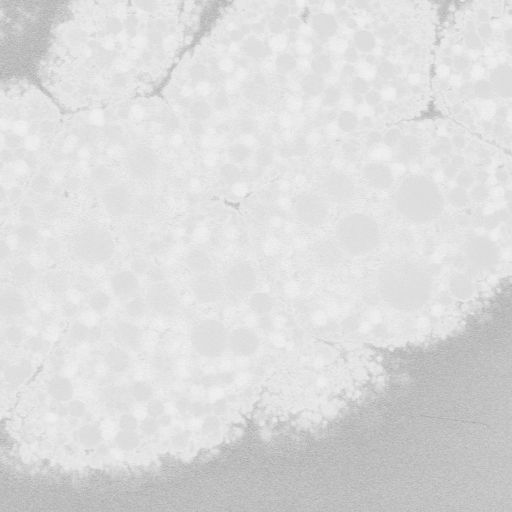

Supplement: Supplementary file 4 — Source Data [file 41467_2024_45576_MOESM4_ESM.zip › Main_Figures/Fig_6 fatbody images/Lys.tif]

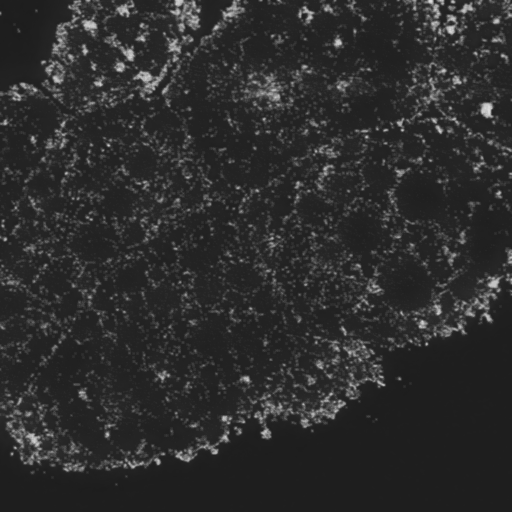

Supplement: Supplementary file 4 — Source Data [file 41467_2024_45576_MOESM4_ESM.zip › Main_Figures/Fig_6 fatbody images/PE.tif]

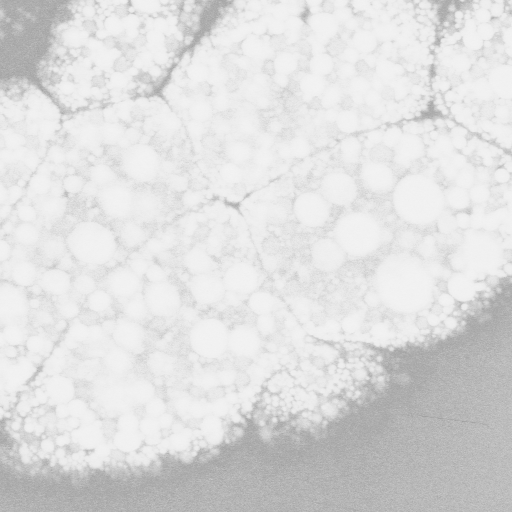

Supplement: Supplementary file 4 — Source Data [file 41467_2024_45576_MOESM4_ESM.zip › Main_Figures/Fig_6 fatbody images/TAG.tif]

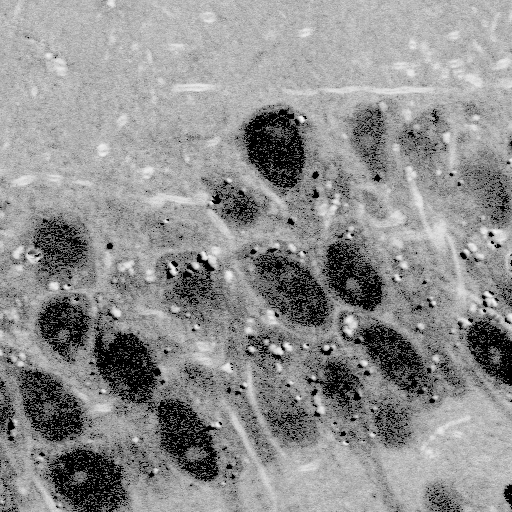

Supplement: Supplementary file 4 — Source Data [file 41467_2024_45576_MOESM4_ESM.zip › Main_Figures/Fig_7 and S_Fig_9 GCMS/Hippocampus/Old brain-sweep-1/Cardiolipin_convolution_image.tiff]

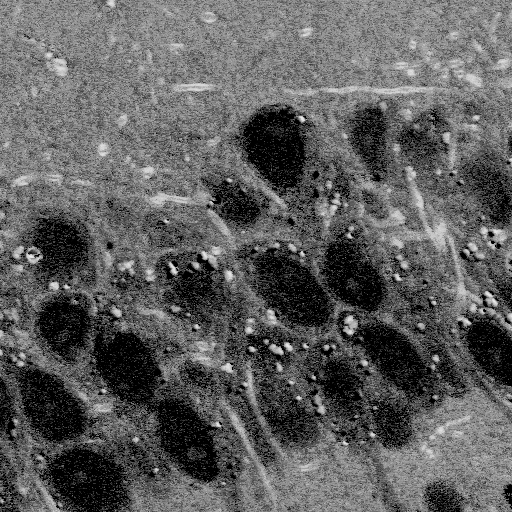

Supplement: Supplementary file 4 — Source Data [file 41467_2024_45576_MOESM4_ESM.zip › Main_Figures/Fig_7 and S_Fig_9 GCMS/Hippocampus/Old brain-sweep-1/CDP_DG_convolution_image.tiff]

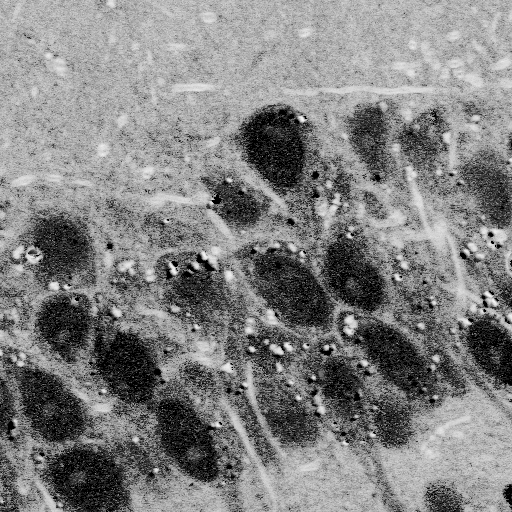

Supplement: Supplementary file 4 — Source Data [file 41467_2024_45576_MOESM4_ESM.zip › Main_Figures/Fig_7 and S_Fig_9 GCMS/Hippocampus/Old brain-sweep-1/Cholesterol_convolution_image.tiff]

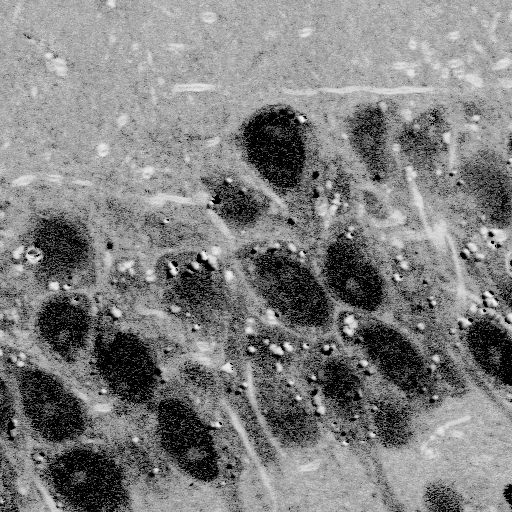

Supplement: Supplementary file 4 — Source Data [file 41467_2024_45576_MOESM4_ESM.zip › Main_Figures/Fig_7 and S_Fig_9 GCMS/Hippocampus/Old brain-sweep-1/Cholesterol_ester_convolution_image.tiff]

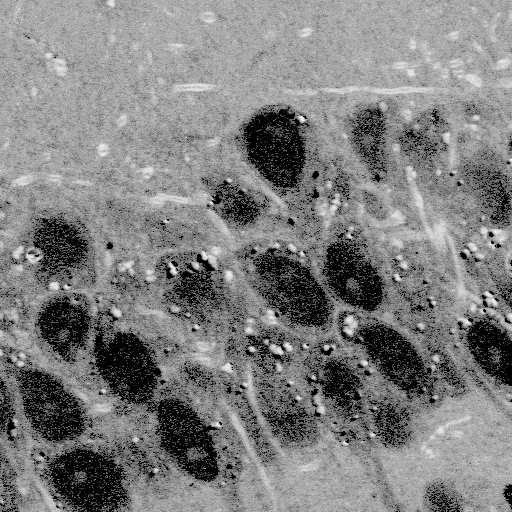

Supplement: Supplementary file 4 — Source Data [file 41467_2024_45576_MOESM4_ESM.zip › Main_Figures/Fig_7 and S_Fig_9 GCMS/Hippocampus/Old brain-sweep-1/dsgPI_convolution_image.tiff]

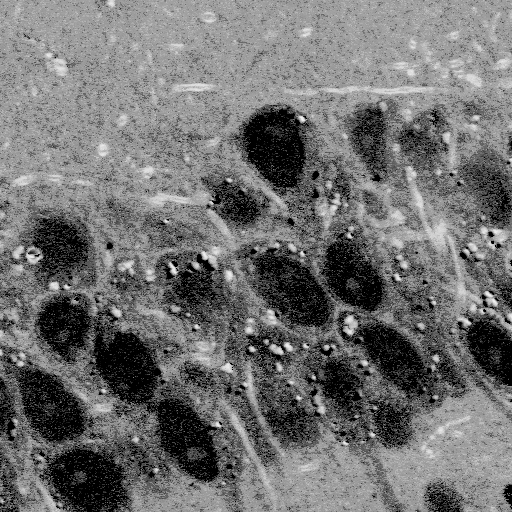

Supplement: Supplementary file 4 — Source Data [file 41467_2024_45576_MOESM4_ESM.zip › Main_Figures/Fig_7 and S_Fig_9 GCMS/Hippocampus/Old brain-sweep-1/LaPG_convolution_image.tiff]

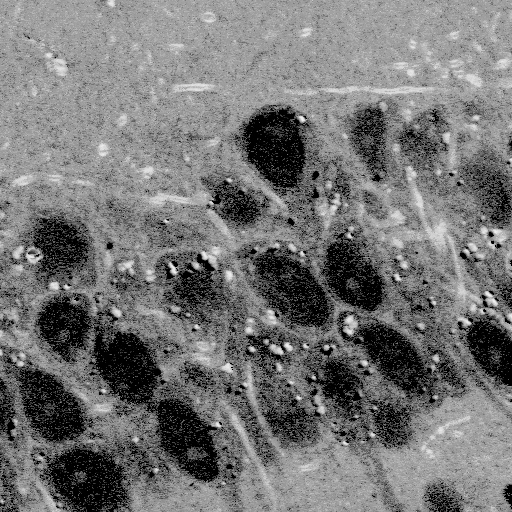

Supplement: Supplementary file 4 — Source Data [file 41467_2024_45576_MOESM4_ESM.zip › Main_Figures/Fig_7 and S_Fig_9 GCMS/Hippocampus/Old brain-sweep-1/LaPI_convolution_image.tiff]

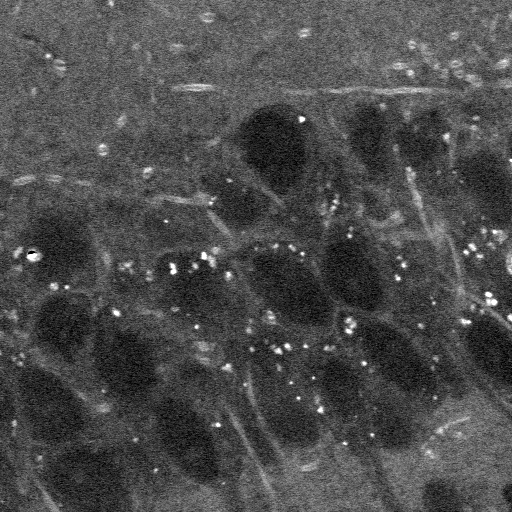

Supplement: Supplementary file 4 — Source Data [file 41467_2024_45576_MOESM4_ESM.zip › Main_Figures/Fig_7 and S_Fig_9 GCMS/Hippocampus/Old brain-sweep-1/lipid_channel.tiff]

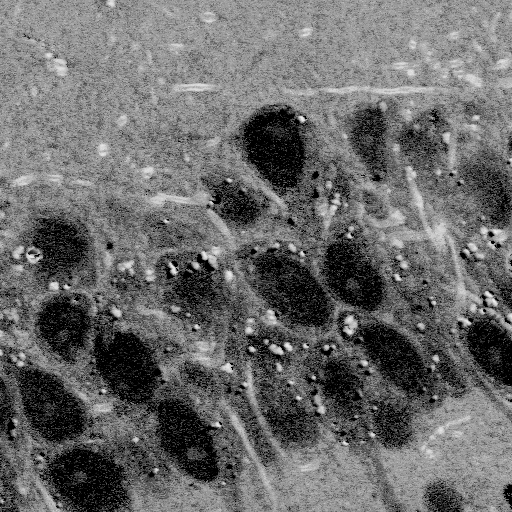

Supplement: Supplementary file 4 — Source Data [file 41467_2024_45576_MOESM4_ESM.zip › Main_Figures/Fig_7 and S_Fig_9 GCMS/Hippocampus/Old brain-sweep-1/LPA_convolution_image.tiff]

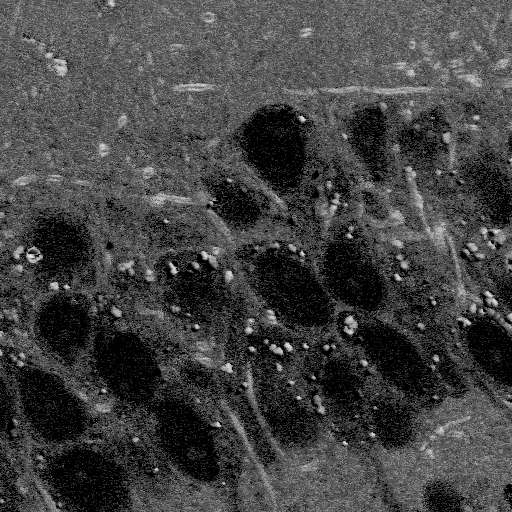

Supplement: Supplementary file 4 — Source Data [file 41467_2024_45576_MOESM4_ESM.zip › Main_Figures/Fig_7 and S_Fig_9 GCMS/Hippocampus/Old brain-sweep-1/Lyso_PA_convolution_image.tiff]

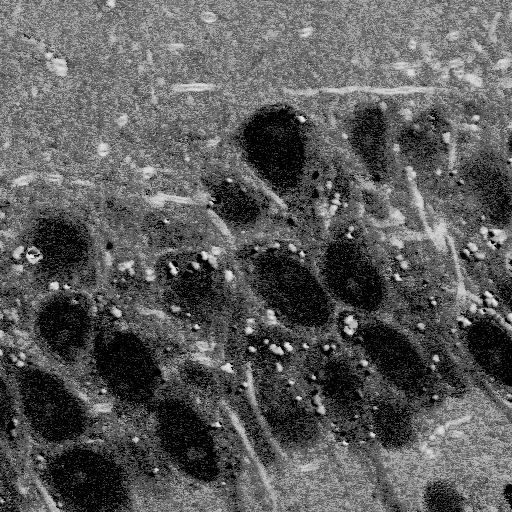

Supplement: Supplementary file 4 — Source Data [file 41467_2024_45576_MOESM4_ESM.zip › Main_Figures/Fig_7 and S_Fig_9 GCMS/Hippocampus/Old brain-sweep-1/Lysyl_DG_convolution_image.tiff]

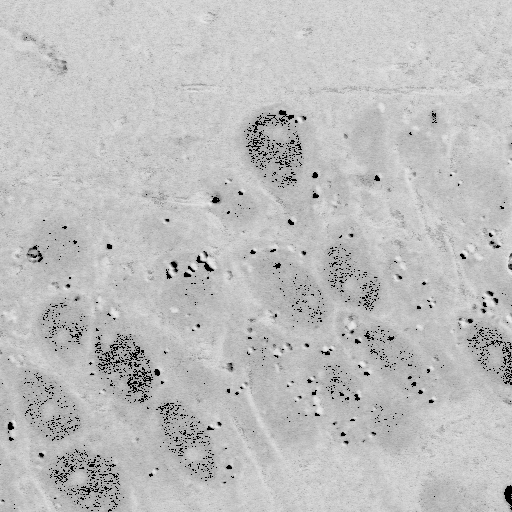

Supplement: Supplementary file 4 — Source Data [file 41467_2024_45576_MOESM4_ESM.zip › Main_Figures/Fig_7 and S_Fig_9 GCMS/Hippocampus/Old brain-sweep-1/MUT_convolution_image.tiff]

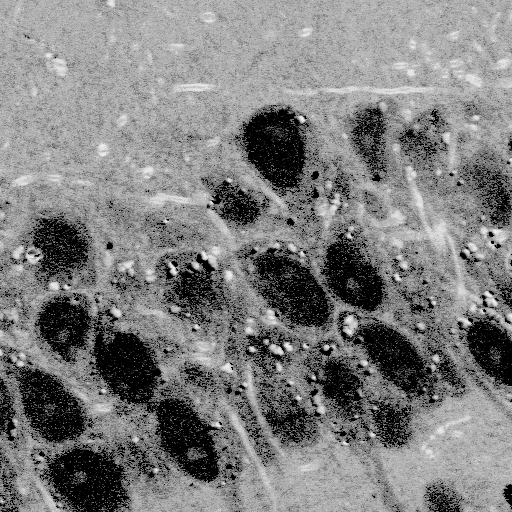

Supplement: Supplementary file 4 — Source Data [file 41467_2024_45576_MOESM4_ESM.zip › Main_Figures/Fig_7 and S_Fig_9 GCMS/Hippocampus/Old brain-sweep-1/PC_convolution_image.tiff]

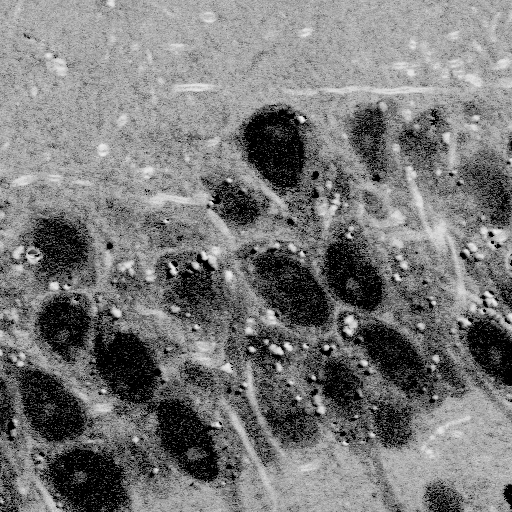

Supplement: Supplementary file 4 — Source Data [file 41467_2024_45576_MOESM4_ESM.zip › Main_Figures/Fig_7 and S_Fig_9 GCMS/Hippocampus/Old brain-sweep-1/PE_convolution_image.tiff]

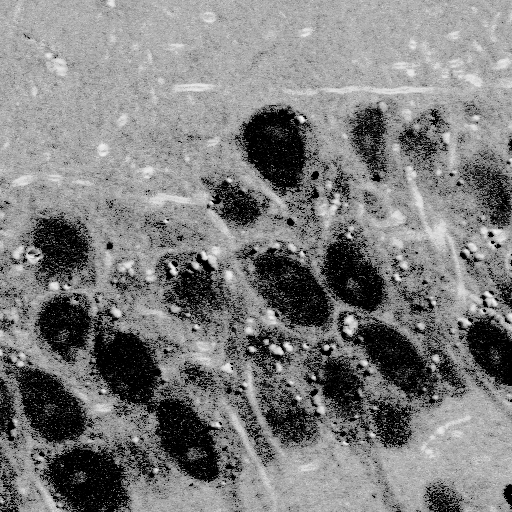

Supplement: Supplementary file 4 — Source Data [file 41467_2024_45576_MOESM4_ESM.zip › Main_Figures/Fig_7 and S_Fig_9 GCMS/Hippocampus/Old brain-sweep-1/Phosphatidylcholine_convolution_image.tiff]

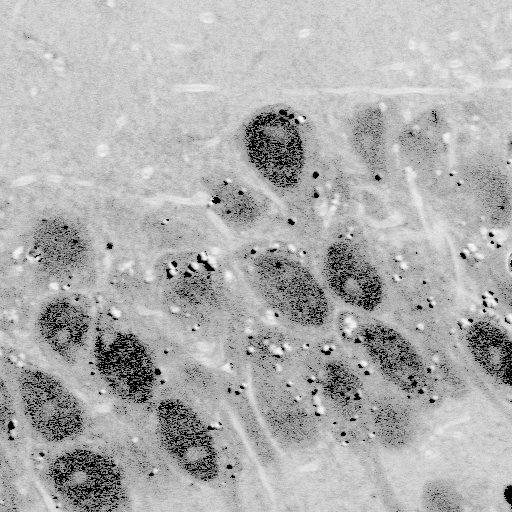

Supplement: Supplementary file 4 — Source Data [file 41467_2024_45576_MOESM4_ESM.zip › Main_Figures/Fig_7 and S_Fig_9 GCMS/Hippocampus/Old brain-sweep-1/PLS_convolution_image.tiff]

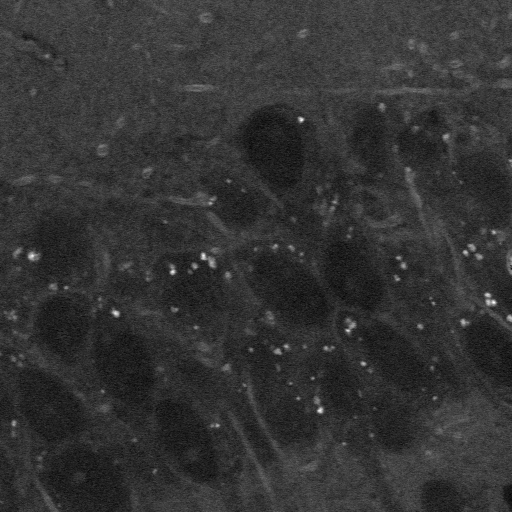

Supplement: Supplementary file 4 — Source Data [file 41467_2024_45576_MOESM4_ESM.zip › Main_Figures/Fig_7 and S_Fig_9 GCMS/Hippocampus/Old brain-sweep-1/protein_channel.tiff]

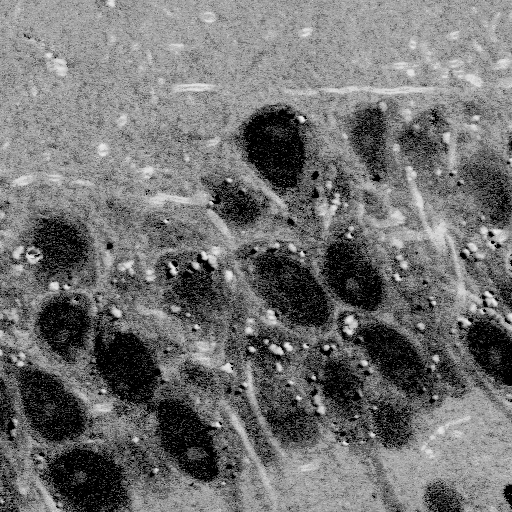

Supplement: Supplementary file 4 — Source Data [file 41467_2024_45576_MOESM4_ESM.zip › Main_Figures/Fig_7 and S_Fig_9 GCMS/Hippocampus/Old brain-sweep-1/PS_convolution_image.tiff]

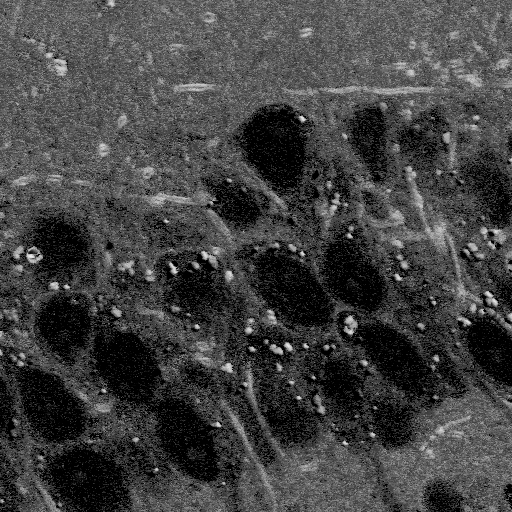

Supplement: Supplementary file 4 — Source Data [file 41467_2024_45576_MOESM4_ESM.zip › Main_Figures/Fig_7 and S_Fig_9 GCMS/Hippocampus/Old brain-sweep-1/Sphingosine_convolution_image.tiff]

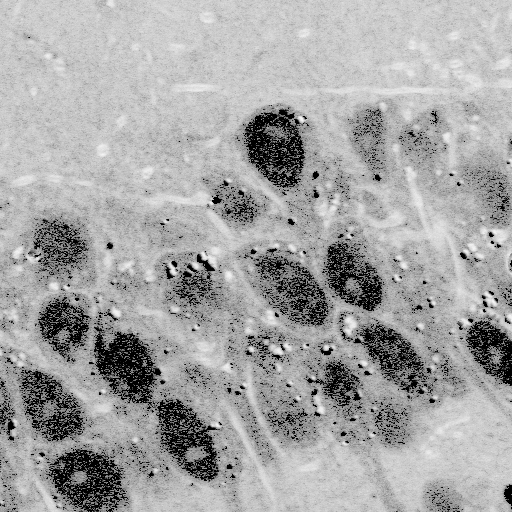

Supplement: Supplementary file 4 — Source Data [file 41467_2024_45576_MOESM4_ESM.zip › Main_Figures/Fig_7 and S_Fig_9 GCMS/Hippocampus/Old brain-sweep-1/TAG_convolution_image.tiff]

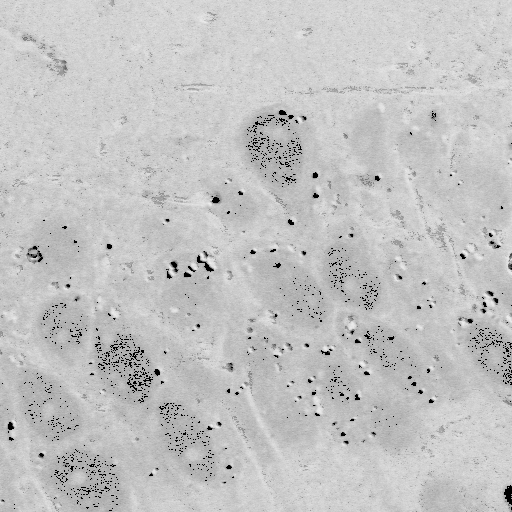

Supplement: Supplementary file 4 — Source Data [file 41467_2024_45576_MOESM4_ESM.zip › Main_Figures/Fig_7 and S_Fig_9 GCMS/Hippocampus/Old brain-sweep-1/WT_convolution_image.tiff]

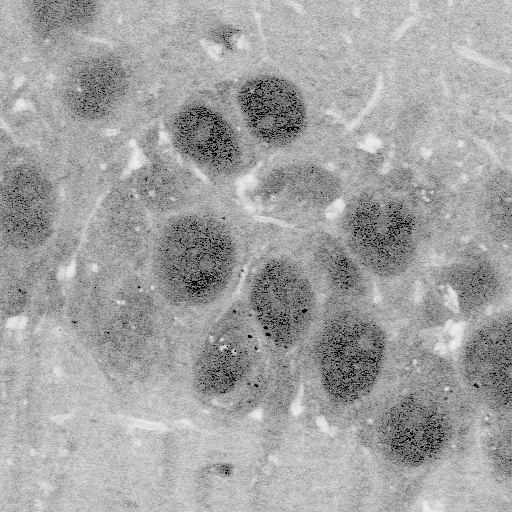

Supplement: Supplementary file 4 — Source Data [file 41467_2024_45576_MOESM4_ESM.zip › Main_Figures/Fig_7 and S_Fig_9 GCMS/Hippocampus/Young brain-sweep-1/Cardiolipin_convolution_image.tiff]

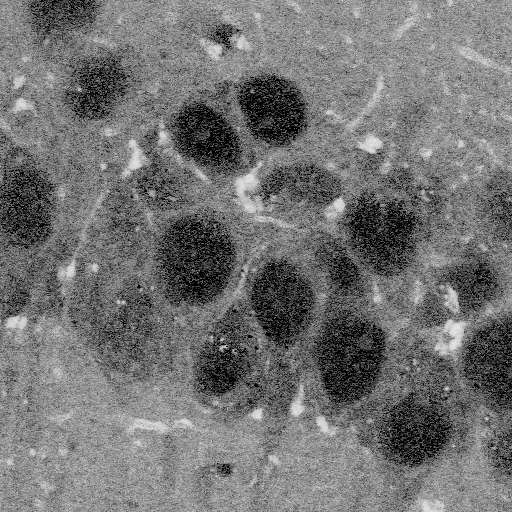

Supplement: Supplementary file 4 — Source Data [file 41467_2024_45576_MOESM4_ESM.zip › Main_Figures/Fig_7 and S_Fig_9 GCMS/Hippocampus/Young brain-sweep-1/CDP_DG_convolution_image.tiff]

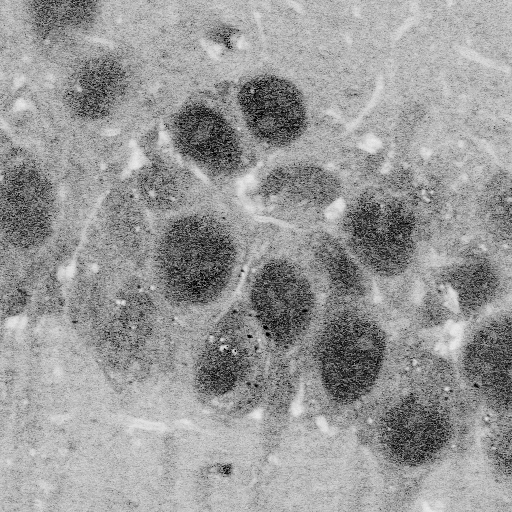

Supplement: Supplementary file 4 — Source Data [file 41467_2024_45576_MOESM4_ESM.zip › Main_Figures/Fig_7 and S_Fig_9 GCMS/Hippocampus/Young brain-sweep-1/Cholesterol_convolution_image.tiff]

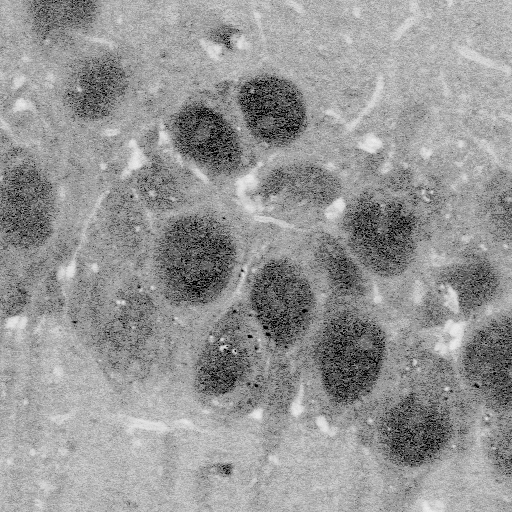

Supplement: Supplementary file 4 — Source Data [file 41467_2024_45576_MOESM4_ESM.zip › Main_Figures/Fig_7 and S_Fig_9 GCMS/Hippocampus/Young brain-sweep-1/Cholesterol_ester_convolution_image.tiff]

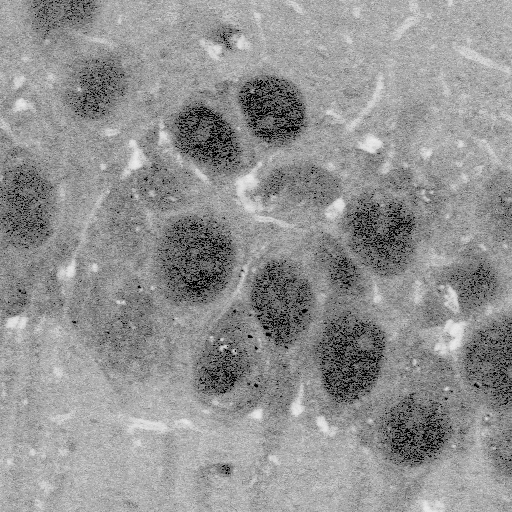

Supplement: Supplementary file 4 — Source Data [file 41467_2024_45576_MOESM4_ESM.zip › Main_Figures/Fig_7 and S_Fig_9 GCMS/Hippocampus/Young brain-sweep-1/dsgPI_convolution_image.tiff]

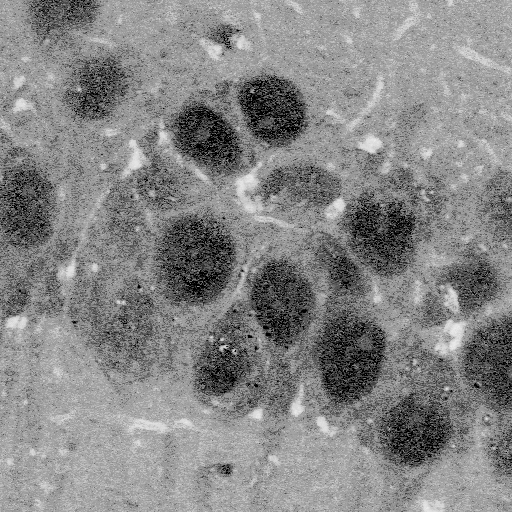

Supplement: Supplementary file 4 — Source Data [file 41467_2024_45576_MOESM4_ESM.zip › Main_Figures/Fig_7 and S_Fig_9 GCMS/Hippocampus/Young brain-sweep-1/LaPG_convolution_image.tiff]

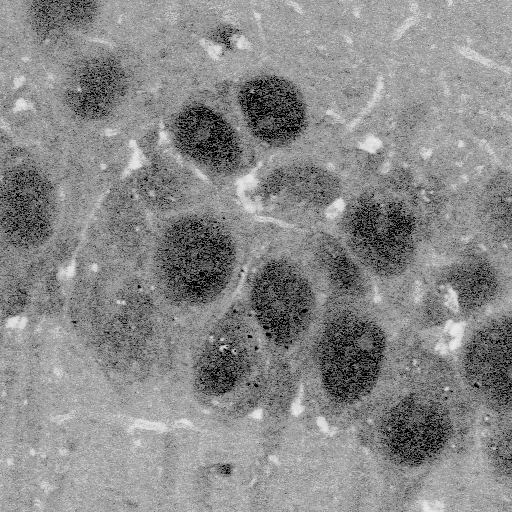

Supplement: Supplementary file 4 — Source Data [file 41467_2024_45576_MOESM4_ESM.zip › Main_Figures/Fig_7 and S_Fig_9 GCMS/Hippocampus/Young brain-sweep-1/LaPI_convolution_image.tiff]

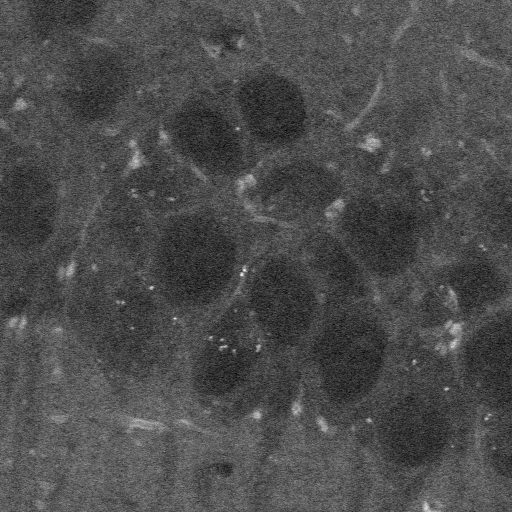

Supplement: Supplementary file 4 — Source Data [file 41467_2024_45576_MOESM4_ESM.zip › Main_Figures/Fig_7 and S_Fig_9 GCMS/Hippocampus/Young brain-sweep-1/lipid_channel.tiff]

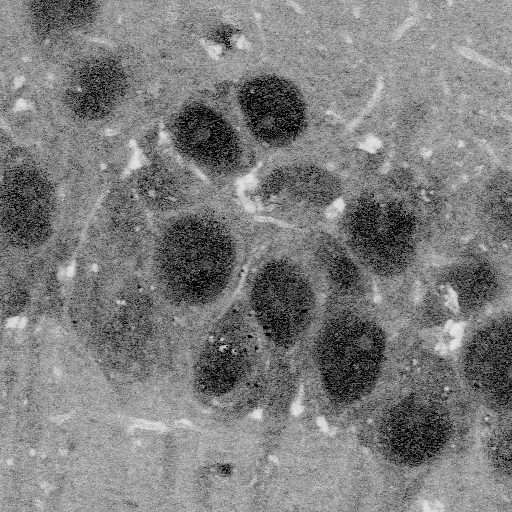

Supplement: Supplementary file 4 — Source Data [file 41467_2024_45576_MOESM4_ESM.zip › Main_Figures/Fig_7 and S_Fig_9 GCMS/Hippocampus/Young brain-sweep-1/LPA_convolution_image.tiff]

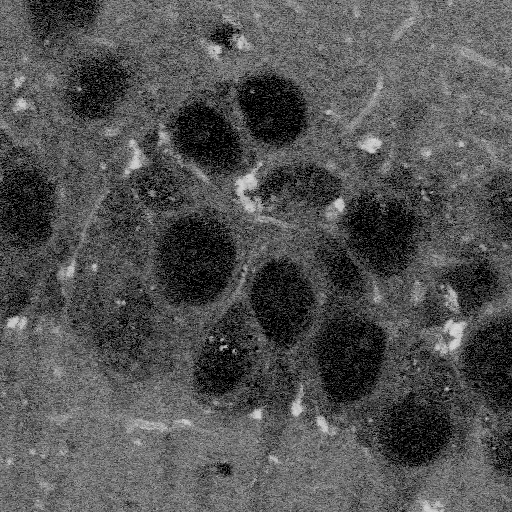

Supplement: Supplementary file 4 — Source Data [file 41467_2024_45576_MOESM4_ESM.zip › Main_Figures/Fig_7 and S_Fig_9 GCMS/Hippocampus/Young brain-sweep-1/Lyso_PA_convolution_image.tiff]

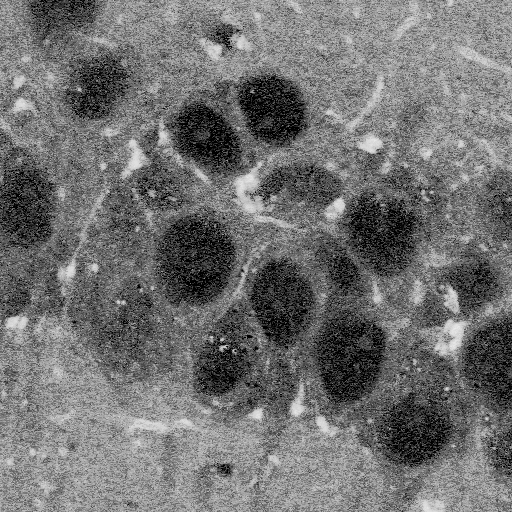

Supplement: Supplementary file 4 — Source Data [file 41467_2024_45576_MOESM4_ESM.zip › Main_Figures/Fig_7 and S_Fig_9 GCMS/Hippocampus/Young brain-sweep-1/Lysyl_DG_convolution_image.tiff]

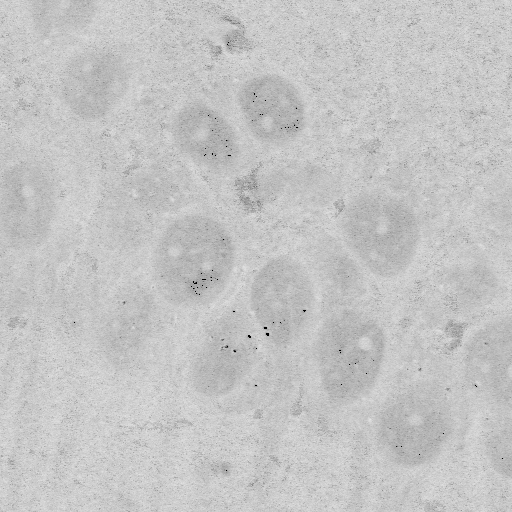

Supplement: Supplementary file 4 — Source Data [file 41467_2024_45576_MOESM4_ESM.zip › Main_Figures/Fig_7 and S_Fig_9 GCMS/Hippocampus/Young brain-sweep-1/MUT_convolution_image.tiff]

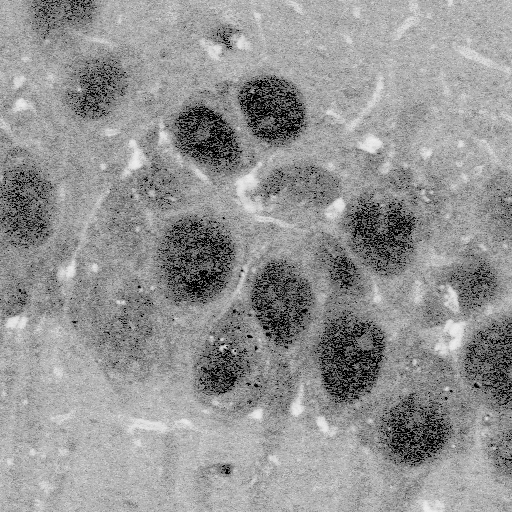

Supplement: Supplementary file 4 — Source Data [file 41467_2024_45576_MOESM4_ESM.zip › Main_Figures/Fig_7 and S_Fig_9 GCMS/Hippocampus/Young brain-sweep-1/PC_convolution_image.tiff]

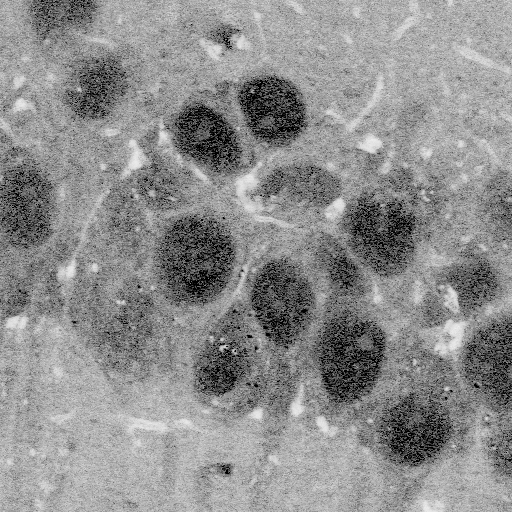

Supplement: Supplementary file 4 — Source Data [file 41467_2024_45576_MOESM4_ESM.zip › Main_Figures/Fig_7 and S_Fig_9 GCMS/Hippocampus/Young brain-sweep-1/PE_convolution_image.tiff]

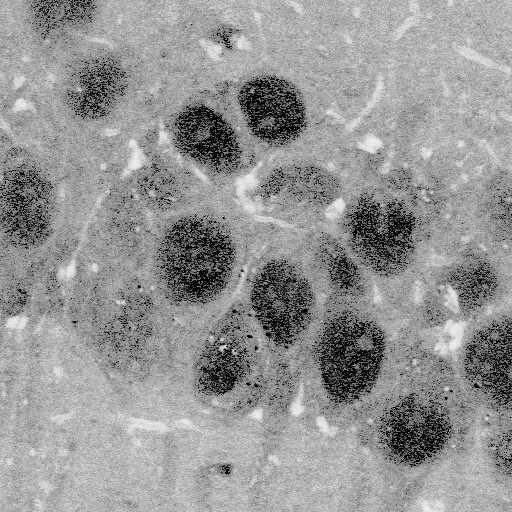

Supplement: Supplementary file 4 — Source Data [file 41467_2024_45576_MOESM4_ESM.zip › Main_Figures/Fig_7 and S_Fig_9 GCMS/Hippocampus/Young brain-sweep-1/Phosphatidylcholine_convolution_image.tiff]

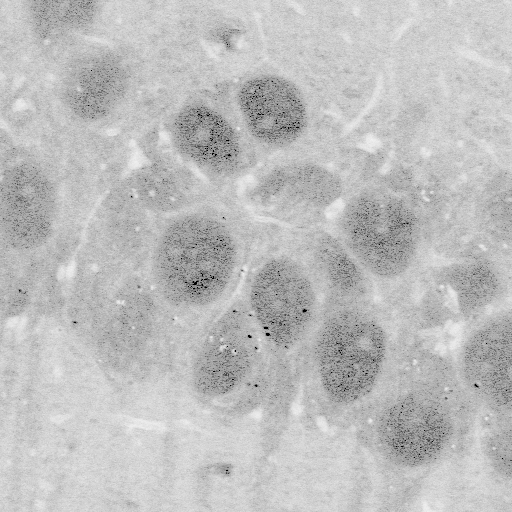

Supplement: Supplementary file 4 — Source Data [file 41467_2024_45576_MOESM4_ESM.zip › Main_Figures/Fig_7 and S_Fig_9 GCMS/Hippocampus/Young brain-sweep-1/PLS_convolution_image.tiff]

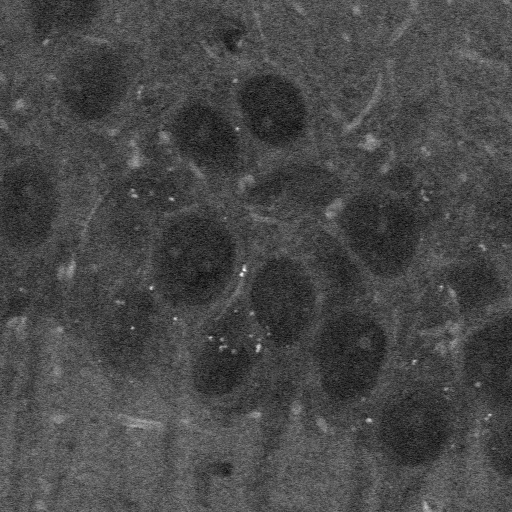

Supplement: Supplementary file 4 — Source Data [file 41467_2024_45576_MOESM4_ESM.zip › Main_Figures/Fig_7 and S_Fig_9 GCMS/Hippocampus/Young brain-sweep-1/protein_channel.tiff]

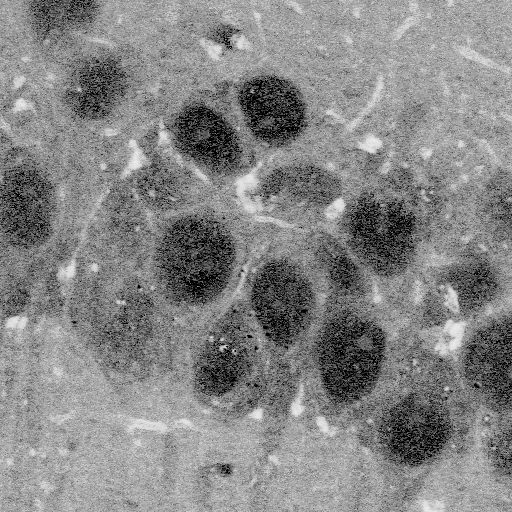

Supplement: Supplementary file 4 — Source Data [file 41467_2024_45576_MOESM4_ESM.zip › Main_Figures/Fig_7 and S_Fig_9 GCMS/Hippocampus/Young brain-sweep-1/PS_convolution_image.tiff]

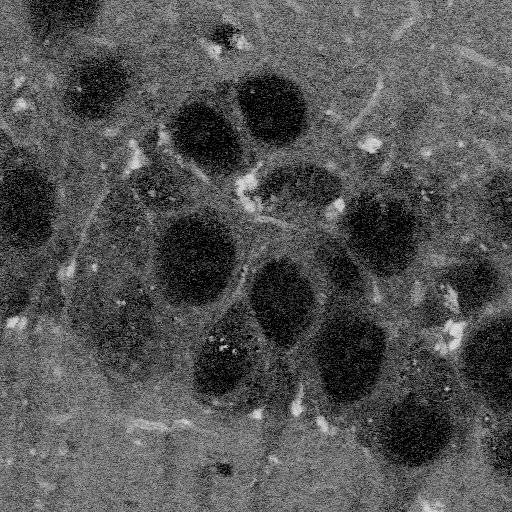

Supplement: Supplementary file 4 — Source Data [file 41467_2024_45576_MOESM4_ESM.zip › Main_Figures/Fig_7 and S_Fig_9 GCMS/Hippocampus/Young brain-sweep-1/Sphingosine_convolution_image.tiff]

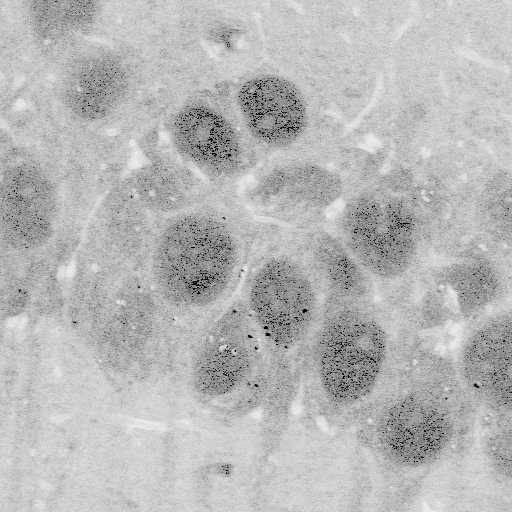

Supplement: Supplementary file 4 — Source Data [file 41467_2024_45576_MOESM4_ESM.zip › Main_Figures/Fig_7 and S_Fig_9 GCMS/Hippocampus/Young brain-sweep-1/TAG_convolution_image.tiff]

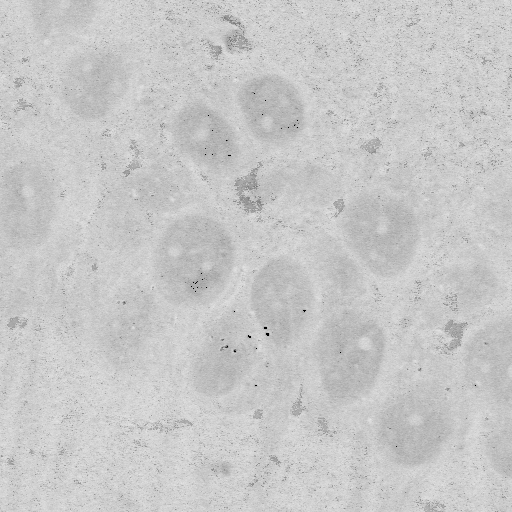

Supplement: Supplementary file 4 — Source Data [file 41467_2024_45576_MOESM4_ESM.zip › Main_Figures/Fig_7 and S_Fig_9 GCMS/Hippocampus/Young brain-sweep-1/WT_convolution_image.tiff]
